# Redox properties of Cys_2_His_2_ and Cys_4_ zinc fingers determined by electrospray ionization mass spectrometry

**DOI:** 10.1002/2211-5463.12422

**Published:** 2018-04-25

**Authors:** Julia Smirnova, Ekaterina Kabin, Vello Tõugu, Peep Palumaa

**Affiliations:** ^1^ Department of Chemistry and Biotechnology Tallinn University of Technology Estonia

**Keywords:** electrospray ionization mass spectrometry, ESI‐MS, midpoint redox potential, zinc finger

## Abstract

Zinc finger (ZF) protein motifs, stabilized by binding of Zn(II), typically function as interaction modules that bind nucleic acids, proteins and other molecules. The elucidation of the redox states of ZF proteins in cellular conditions, which depend on their midpoint redox potentials, is important for understanding of ZF functioning. In the present study we determined the midpoint redox potentials for representatives of Cys_2_His_2_ and Cys_4_ types of ZF proteins in apo and Zn(II)‐bound forms using electrospray ionization mass spectrometry. The midpoint redox potentials of the apo forms of Cys_2_His_2_ and Cys_4_ ZF proteins were −326 and −365 mV (pH 7.5), respectively. These values are close to the cytosolic redox potential of approx. −350 mV (pH 7.5) and thus we can conclude that the apo form of Cys_2_His_2_‐type ZF proteins is predominantly reduced but apo forms of Cys_4_‐type ZF proteins should be substantially oxidized in the cytoplasm. As expected, Zn(II) binding stabilized the reduced forms of both ZF proteins: the corresponding redox potential values were −284 and −301 mV, respectively. Consequently, binding of Zn(II) ions to ZF motifs can act as a sensitive switch that activates the functioning of the ZF motifs within the cell, and also protects them from oxidation and can function as part of a redox‐sensitive regulation mechanism of cellular functions.

**Abbreviations**
BME_ox_: oxidized β‐mercaptoethanol
BME: β‐mercaptoethanol
DTT: dithiotreitol
DTT_ox_: oxidized dithiotreitol
ESI: electrospray ionization
GSH: glutathione
GSSG: oxidized glutathione
MDM2: murine double minute 2
MS: mass spectrometry
Q‐TOF: quadrupole time of flight
ZF: zinc finger
ZNF268: DNA‐binding ZF protein 268

Zinc finger (ZF) motifs consist of small protein domains (typically containing 30–40 amino acid residues) that are ubiquitous in eukaryotes 1 and involved as interaction modules for recognition of nucleic acids, proteins and small molecules 2, 3, 4. ZFs exist as independent entities or as domains in larger proteins and their tertiary structure is usually stabilized by Zn(II) ions tetrahedrally coordinated by at least two Cys thiolates in combination with His 4. Historically, the ZF motifs were classified according to the coordinating groups in the Zn(II)‐binding center and the most common types are Cys_2_His_2_ and Cys_4_ 5, 6.

The redox reactivity of ZF motifs is determined by Cys thiol groups, which are the only versatile redox‐active groups in proteins. Thiol groups in ZF can exist in three major states: thiol‐thiolate, Zn(II)‐bound thiolate and oxidized disulfide. The shift of the redox balance in the cells or cell compartments to the oxidative side may result in oxidation of Cys residues to disulfide and the release of Zn(II) ions with concomitant loss of ZF functions 7, 8, 9, 10, 11. However, the redox properties of ZFs, which determine their redox state in the cellular redox environment, have not been characterized quantitatively. Redox properties of the environment are characterized by the reduction potential (redox potential, oxidation–reduction potential, pE, *E*_h_), reflecting the ability of chemical species in the particular environment to acquire electrons and thereby be reduced 12. The redox potentials in different cellular compartments and the extracellular space are maintained at different values by several redox pairs. The most essential redox pairs in eukaryotes are glutathione (GSH)–oxidized glutathione (GSSG), cysteine–cystine and thioredoxin pairs 13, 14. The reducing ability, determined by reduction potential of cellular compartments, decreases in the order mitochondria ˃ nuclei ˃ cytoplasm ˃ endoplasmic reticulum ˃ extracellular space 15, 16, and depends on the cell cycle, stress conditions and multiple other factors 14. The redox potential value of the environment where half of the particular residue(s) in the protein are oxidized is usually referred to as a midpoint redox potential and denoted as *E*_m_.

The aim of the current study was a quantitative description of redox properties of ZFs through determination of their *E*_m_ values, which helps in understanding functioning and redox regulation of ZFs. Namely, by comparing *E*_m_ values with the cellular redox potential values we can conclude in which redox state ZFs exist under changing redox conditions within cells. For our studies we have chosen two representatives of ZF proteins with different Zn(II)‐coordinating ligands. The first one, ZF‐1, (PDB ID http://www.rcsb.org/pdb/search/structidSearch.do?structureId=2el5) is the 18th Cys_2_His_2_‐type ZF domain of human DNA‐binding ZF protein 268 (ZNF268). ZNF268 is involved in cell development during embryogenesis and the postnatal period in higher mammals. Disorders in ZNF268 expression and processing may cause cancer and leukemia, but not all functions of ZNF268 are yet known. The second protein, ZF‐2, (PDB ID http://www.rcsb.org/pdb/search/structidSearch.do?structureId=2c6a) is the Cys_4_‐type ZF domain of a ubiquitin E3 ligase murine double minute 2 protein, MDM2. MDM2 plays a key role in tumorigenesis through p53 tumor suppressor inactivation 17, 18, 19.

Electrospray ionization mass spectrometry (ESI‐MS) has proved to be a suitable method for determination of the redox properties of various peptides and small proteins *in vitro* 20, 21, 22, 23, and the results obtained by ESI‐MS are in line with the results obtained by other techniques 22, 24. An advantage of the ESI‐MS approach is the ability to directly identify and quantify all redox forms of proteins and protein–metal complexes as well as labile intermediates and adducts simultaneously 20.

In the present study the midpoint redox potentials for representatives of Cys_2_His_2_ (ZF‐1) and Cys_4_ (ZF‐2) types of ZF proteins in apo and Zn(II)‐bound forms were determined using ESI‐MS. Based on the results obtained we can conclude that the apo form of the Cys_2_His_2_ type of ZF proteins is predominantly reduced but the apo form of the Cys_4_ type of ZF proteins should be substantially oxidized in the cytoplasm, and Zn(II) ions protect both ZF proteins from oxidation and ensure their functioning.

## Materials and methods

### Chemicals

Human ZF peptides ZF‐1 (GENPYECSECGKAFNRKDQLISHQRTHAGES) and ZF‐2 (SFEEDPEISLADYWKCTSCNEMNPPLPSHCNRCWALRENWLPEDKG), of purity 95%, were obtained from Peptide 2.0 Inc. (Chantilly, VA, USA); dithiotreitol (DTT); β‐mercaptoethanol (BME), oxidized BME (BME_ox_) and ammonium hydroxide solution (25%) were from Sigma‐Aldrich (St Louis, MO, USA); Zn(II) acetate was from Scharlau (Barcelona, Spain); and formic acid was from Riedel‐de Häen (Seelze, Germany). Oxidized DTT (DTT_ox_) was synthesized from reduced DTT according to the protocol described in 25. Ultrapure water (EASYpure^®^II UV/UF ultrapure water system, Barnstead, Lake Balboa, CA, USA) was used for preparation of solutions.

### Kinetics of apo‐ZF reduction and oxidation

Weighted solid samples of ZF‐1 and ZF‐2 were dissolved in 75 mm ammonium formate, pH 7.5 (argon purged), to prepare 200 μm stock solutions. The volatile compounds BME and BME_ox_ were used for redox buffering since this pair is more suitable for ESI‐MS measurements than biological system containing GSH/GSSG 23.

Apo‐ZF‐1 was completely oxidized in the stock solution. For the kinetic measurements of apo‐ZF‐1 reduction, 5 μm peptide was incubated with 25 mm BME at 25 °C; 50 μL aliquots were taken at different time points (2, 30, 60, 120 and 180 min) and injected by a syringe pump into a QSTAR Elite ESI quadrupole time of flight (Q‐TOF) mass spectrometer (Applied Biosystems, Foster City, CA, USA) at flow rate 10 μL·min^−1^.

Apo‐ZF‐2 was partially oxidized in the stock solution. In order to obtain information about the air oxidation of the diluted ZF‐2, 5 and 10 μm apo‐ZF‐2 samples were incubated in 75 mm ammonium formate buffer, pH 7.5 at 25 °C; 50 μL aliquots of the sample were injected into the mass spectrometer as described for apo‐ZF‐1. For kinetic measurements, 5 μm apo‐ZF‐2 samples containing 50 mm BME as a reducing agent or 25 mm BME_ox_ as an oxidizing agent were incubated at 40 °C for 3 h; 50 μL aliquots of each reaction mixture were injected into the mass spectrometer as described above. All mass spectra were recorded in the positive mode for 2–3 min in the *m*/*z* region 700–2000 Da. The working parameters of the instrument were: ion spray voltage, 5.5 kV; source gas, 55 L·min^−1^; curtain gas, 20 L·min^−1^; declustering potential 1, 60 V; declustering potential 2, 15 V; focusing potential, 320 V; ion release delay, 6; ion release width, 5; ion energy, 2; channel electron multiplier, 2450V; detector voltage, 2.3 kV. The spectra were deconvoluted and analyzed with the bioanalyst 2.0 program (Applied Biosystems).

All data were analyzed by using origin 8.0 (OriginLab Corp., Northampton, MA, USA).

### Determination of the midpoint redox potential for apo‐ZFs

For the measurements of the midpoint redox potentials of the apo forms of the peptides, 5 μm of apo‐peptides were incubated in BME/BME_ox_ redox buffers with different redox potential values generated by different ratio of BME/BME_ox_ (0–100%) in order to achieve the redox equilibrium between the protein forms (ZF‐1_0S–S_ and ZF‐1_1S–S_; ZF‐2_0S–S_ and ZF‐2_2S–S_). Argon‐purged 75 mm ammonium formate, pH 7.5, was used in all experiments.

The equilibrium ratios of apo‐peptides were determined by ESI‐MS after 30 min incubation in BME/BME_ox_ redox buffers with total concentrations ([BME] + 2[BME_ox_]) equal to 25 mm at 25 °C in the case of ZF‐1 and 50 mm at 40 °C in the case of ZF‐2. Conditions for ESI‐MS measurements were the same as described above. The relative content of the reduced form of the peptide, referred to as fractional content (*y*), was calculated from the experimentally determined average molecular mass (*m*) of the mixture of reduced and oxidized peptides in different redox buffers taking into account all charge states present in the spectrum by using the following equation:

(1) $$y=m_{C}-m_{\mathrm{ZFS}-S}/x$$

where *x* is the difference in molecular masses of reduced ZF and fully oxidized ZF peptides (*x* = 2 Da in the case of ZF‐1 and *x* = 4 Da in the case of ZF‐2), *m*_ZFS–S_ is the calculated molecular mass of the fully oxidized peptide, and *m*_C_ is the experimentally determined average molecular mass of the mixture of reduced and oxidized peptides under particular redox conditions.

The redox potential of the buffer was created by varying the ratio of reduced and oxidized forms of BME. Corresponding redox potential values were calculated according to the Nernst equation:

(2) $$E^{\prime }=E_{0}^{\prime }\left(\text{BME}\right)-\frac{\mathrm{RT}}{\mathrm{nF}}\ln\frac{\left[{\text{BME}}^{2}\right]}{\left[{\text{BME}}_{\mathrm{ox}}\right]}$$

where *E*ʹ_0_(BME) = −0.231 V (pH 7.0 and 25 °C) 26, *R* is the gas constant (8.315 J·K^−1^·mol^−1^), *n* is the number of electrons transferred in the reaction, and *F* is the Faraday constant (9.6485 × 10^4^ C·mol^−1^). *E*ʹ_0_(BME) values for 40 °C were calculated using Eqn (2) and for pH 7.5 using:

(3) $$E_{\mathrm{pH}}=E_{0}^{\prime }+\left(\text{pH}-{\text{pH}}_{0}\right)\frac{\Delta E}{\Delta \text{pH}}$$

where Δ*E*/ΔpH is equal to −60.1 mV 27.

The midpoint redox potentials for the apo‐ZFs were determined from the dependence of fractional content of fully reduced apo‐peptide (*y*) from the environmental redox potential (*x*) by fitting it to the Boltzmann equation:

(4) $$y=\frac{A_{1}-A_{2}}{1+e^{(x-x_{0})/dx}}+A_{2}$$

where *A*_1_ and *A*_2_ are constants, and *x*_0_ is the midpoint redox potential (*E*_m_). Data were fitted using an *A*_1_ of 0 (initial fractional content of ZF_0S–S_) and an *A*_2_ of 1 (final fractional content of ZF_0S–S_) by using origin 8.0.

### Kinetics of Zn(II) release from Zn_1_ZF‐1 and Zn_1_ZF‐2 with BME_ox_ monitored by ESI‐MS

For determination of the release of Zn(II) ions from the Zn(II)‐loaded forms of the ZF peptides, the mixtures of 5 μm apo‐peptides with 7 μm Zn(II) acetate were incubated with 25 mm BME_ox_ at 25 or 40 °C for 3 h; 50 μL aliquots were injected into the QSTAR Elite ESI‐Q‐TOF mass spectrometer at various time points (2, 30, 60, 120 and 180 min) and the MS spectra were recorded as described above. Fresh stock solution of Zn(II) acetate was prepared before measurements.

### Determination of the midpoint redox potential of Zn_1_ZF‐1 and Zn_1_ZF‐2

For the estimation of the effect of Zn(II) ions on the redox equilibrium of ZF‐1_1S–S_/ZF‐1_0S–S_ and ZF‐2_2S–S/_ZF‐2_0S–S_ pairs, the midpoint redox potential of ZF peptides was determined also in the presence of the freshly prepared 7 μm Zn(II) acetate in the 50 mm BME/BME_ox_ redox buffers. Samples were incubated for 30 min at 25 °C in the case of ZF‐1 and for 3 h at 40 °C in the case of ZF‐2 and ESI‐MS spectra were measured as described above. Fractional content (*y*) of Zn_1_ peptides in the mixture of oxidized apo‐ZF and Zn_1_ZF was determined from areas of MS peaks for oxidized apo‐ZF (*S*_apoZF_) and Zn_1_ZF (*S*_ZnZF_) forms by using the following equation:

(5) $$y=S_{\mathrm{ZnZF}}/\left(S_{\mathrm{apoZF}}+S_{\mathrm{ZnZF}}\right)$$

Midpoint redox potentials of Zn_1_ peptides were determined by fitting the fractional content of Zn_1_ peptides in the mixture of oxidized apo‐ZF and Zn_1_ZF to Eqn (4) as described above.

## Results

### Oxidation and reduction of apo‐ZFs

**Figure 1:**
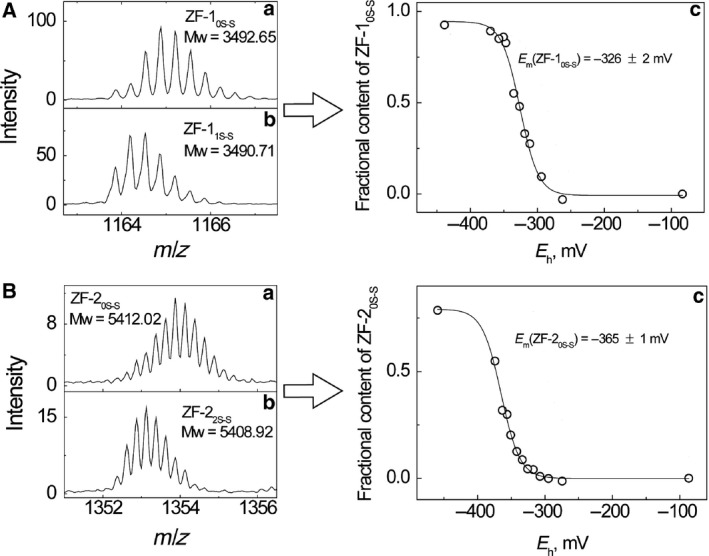
High resolution ESI‐MS spectra of apo‐ZF‐1 (A) and apo‐ZF‐2 (B) incubated with BME or BME_ox_ and determination of redox midpoint potential of apo‐peptides in BME/BME_ox_ redox buffers. Conditions: 5 μm peptide, 75 mm ammonium formate, pH 7.5. +3 charge state is presented for ZF‐1 and +4 for ZF‐2.

Electrospray ionization mass spectrometry spectra of apo‐ZF‐1 consisted of two main peaks with baseline‐resolved isotopic resolution corresponding to charges +3 and +4, which allowed the determination of the average molecular mass of the peptides with high accuracy. The experimentally determined *m* of freshly dissolved ZF‐1 was 3490.71 Da, which corresponded to the *m* of oxidized peptide with a disulfide bond (calculated *m* of ZF‐1_1S–S_ is 3490.78 Da) (Fig. 1Ab). In kinetic experiments apo‐ZF‐1 was reduced by DTT and BME. apo‐ZF‐1 was completely reduced by both reagents within minutes (*t*_1/2_ < 5 min) already at 25 °C (Fig. 1Aa).

Electrospray ionization mass spectrometry spectra of apo‐ZF‐2 displayed two major peaks with charges +4 and +5. The isotopic resolution of the peaks was also at the baseline level. In contrast to apo‐ZF‐1, apo‐ZF‐2 was not fully oxidized: the experimental average *m* of freshly dissolved apo‐ZF‐2 (5411.76 Da) corresponded to the mixture of oxidized and reduced forms of the peptide (calculated *m* of reduced ZF‐2 is 5412.96 Da). Therefore, the kinetics of apo‐ZF‐2 oxidation in the presence of oxidized DTT and BME was also studied. Mixing of apo‐ZF‐2 with 25 mm DTT resulted in almost instant reduction of the peptide, but oxidation at the same concentration of DTT_ox_ was slow and only partial oxidation of apo‐ZF‐2 was achieved after 3 h of incubation at 40 °C. The treatment of apo‐ZF‐2 with 50 mm BME and 25 mm BME_ox_ resulted in fast and complete reduction and oxidation of the apo‐peptide with half‐life <5 min at 40 °C (Fig. 1Ba,b). A small amount of BME adducts were detected in the spectrum (data not shown). The experimentally determined average *m* values of the apo‐ZF‐2 peptide after incubation with reduced and oxidized BME were almost equal to the calculated *m* values of fully reduced (5412.69 Da) and oxidized (5408.87 Da) forms of the peptide, respectively.

Considering the results of kinetic measurements, the determination of the midpoint redox potential of apo‐ZF‐1 was carried out in the presence of 25 mm BME/BME_ox_ redox pair and the samples were equilibrated at 25 °C for 30 min, which was sufficient for reaching an equilibrium. For determination of the midpoint redox potential of ZF‐2, the samples were incubated in 50 mm BME/BME_ox_ at 40 °C for 30 min.

### Midpoint redox potential of apo‐ZFs

The redox potential of apo‐ZF‐1 was determined from the equilibria of oxidized and reduced forms of apo‐ZF‐1 in redox buffers containing BME and BME_ox_ in different ratios at total concentration of [BME] + 2[BME_ox_] of 25 mm. The determined redox potential value, *E*_m_(apo‐ZF‐1), at pH 7.5 was equal to −326 ± 2 mV (Fig. 1Ac). This redox potential value was determined in zinc‐free conditions; no traces of Zn(II) adducts were detected in ESI‐MS spectra even under reducing conditions at pH 7.5 where the affinity of ZF to Zn(II) is the highest.

The midpoint redox potential of apo‐ZF‐2 was determined from the shift in equilibrium of apo‐ZF‐2_2S–S_ and apo‐ZF‐2_0S–S_ in BME/BME_ox_ redox buffers (total concentration of [BME] + 2[BME_ox_] was 50 mm) at pH 7.5 (Fig. 1Bc). A minor peak corresponding to Zn_1_ZF‐2 was present in the ESI‐MS spectra under reducing conditions, which, however, did not disturb the determination of the concentration of reduced and oxidized species. As the preparation of ZF‐2 and the buffer used contained practically no zinc (determined by inductively coupled plasma MS), we concluded that presumably ZF‐2 binds trace amounts of Zn(II) ions from the tubing of the ESI‐MS system. The redox potential value for apo‐ZF‐2_0S–S_ at pH 7.5 was −365 ± 1 mV.

### Release of Zn(II) from Zn_1_ ZF

**Figure 2:**
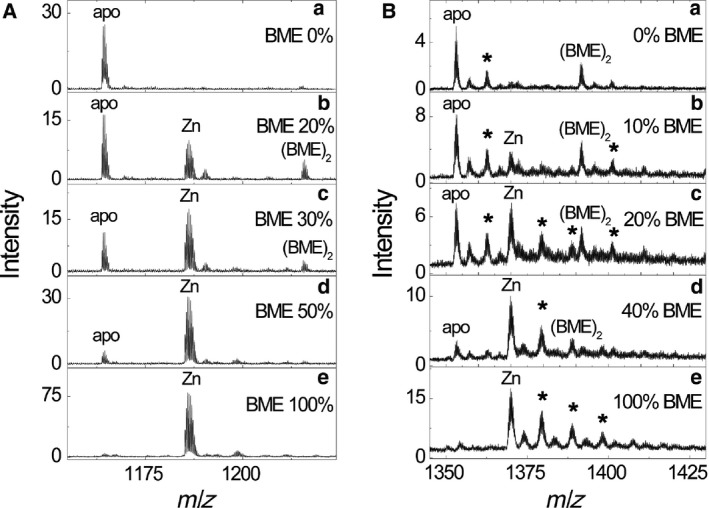
Determination of redox equilibria of Zn_1_ ZF‐1 (A) and Zn_1_ ZF‐2 (B) in 50 mm BME/BME_ox_ redox buffers. Conditions: 5 μm peptide, 7 μm zinc acetate, 75 mm ammonium formate, pH 7.5. Asterisks denote K^+^ adducts. +3 charge state is presented for ZF‐1 and +4 for ZF‐2.

The oxidized form of apo‐ZF‐1 does not bind Zn(II) ions (Fig. 2Aa), but incubation of apo‐ZF‐1 with 25 mm BME in the presence of Zn(II) ions resulted in an immediate appearance of Zn_1_ZF‐1, indicative for binding of Zn(II) ions by reduced peptide form. Figure 2A shows ESI‐MS spectra of Zn_1_ZF‐1 incubated for 30 min in 25 mm BME/BME_ox_ buffers. Apo‐ZF‐1_1S–S_ was the only form present in the oxidizing conditions (Fig. 2Aa), while Zn_1_ZF‐1 was prevalent under reducing conditions (Fig. 2Ae). Under intermediate redox conditions, the ratios of the apo‐ZF‐1_1S–S_ and Zn_1_ZF‐1 is dependent on the redox potential values of the environment determined by the ratio of BME/BME_ox_ (Fig. 2A). A minor peak corresponding to ZF‐1‐(BME)_2_ was also detected at lower BME concentrations (Fig. 2Ab,c). The *m* of the adduct corresponded to the ZF‐1 protein with two BME molecules covalently attached to the Cys residues. The data in Fig. 2A were used for the calculation of the midpoint redox potential for Zn_1_ZF‐1.

**Figure 3:**
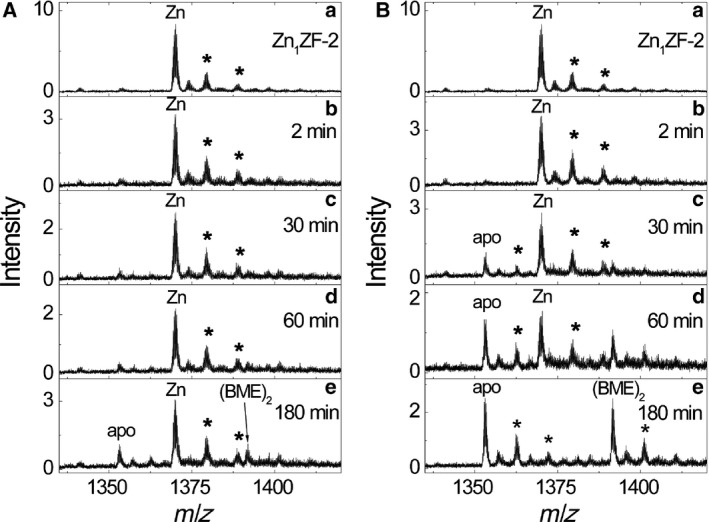
Release of Zn(II) ions from Zn_1_ ZF‐2 in the presence of 25 mm BME_ox_ at 25 °C (A) and 40 °C (B). Conditions: 5 μm apoZF‐2, 7 μm zinc acetate, 75 mm ammonium formate, pH 7.5. Asterisks denote K^+^ adducts. +4 charge state is presented for ZF‐2.

Kinetic studies of ZF‐2 reduction in the presence of 7 μm Zn(II) showed that Zn(II) ions immediately bound to reduced apo‐ZF‐2 at both 25 and 40 °C (Fig. 3Aa,Ba). The incubation of the resulting holo‐ZF‐2 with 25 mm BME_ox_ at 25 °C for 3 h was not sufficient for the complete release of Zn(II) ions as only a minor apo‐ZF‐2_2S–S_ peak was detected in the spectra (Fig. 3Ae). At 40 °C the release of Zn(II) ions from Zn_1_ZF‐2 was more efficient (Fig. 3B): after 3 h of incubation Zn_1_ZF‐2 disappeared and the two major peaks in spectra corresponded to apo‐ZF‐2_2S–S_ and an apo‐ZF‐2‐[BME]_2_ adduct (Fig. 3Be). The average mass of the apo‐ZF‐2 peak (5408.93 Da) was equal to the mass of the fully oxidized peptide. Due to a low reaction rate and incomplete oxidation at 25 °C, the midpoint redox potential of Zn_1_ZF‐2 could be reliably determined only at the elevated temperature of 40 °C.

Up to three K^+^ adducts appeared in the spectrum of ZF‐2 in the presence of Zn(II). During all experiments, the size of the K^+^ adduct peaks remained proportional to all ZF‐2 peaks (apo, Zn(II) and (BME)_2_) and therefore the K^+^ adducts were not taken into account in the calculations. The (BME)_2_ adduct, which appeared only at high concentrations of BME_ox_, was also not taken into account in the calculations.

### Midpoint redox potential of Zn_1_ ZFs in the BME/BME_ox_ redox buffer

**Figure 4:**
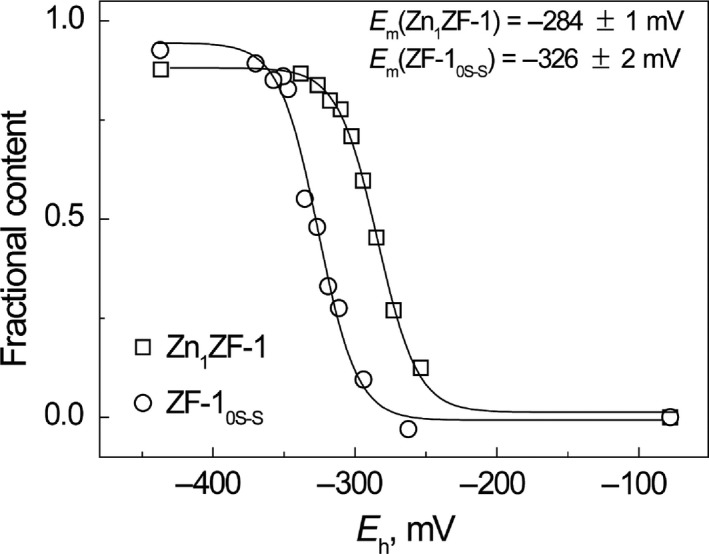
Comparison of redox midpoint potentials of apoZF‐1 and Zn_1_ ZF‐1 in BME/BME_ox_ redox buffers. Fractional content of Zn_1_ ZF‐1 (□) and ZF‐1_0S–S_ (○) at different environmental redox potential values generated by 50 mm BME/BME_ox_ (□) and 25 mm BME/BME_ox_ (○). Conditions: 5 μm ZF‐1, 75 mm ammonium formate, pH 7.5; 25 °C. The lines are fitted curves.

**Figure 5:**
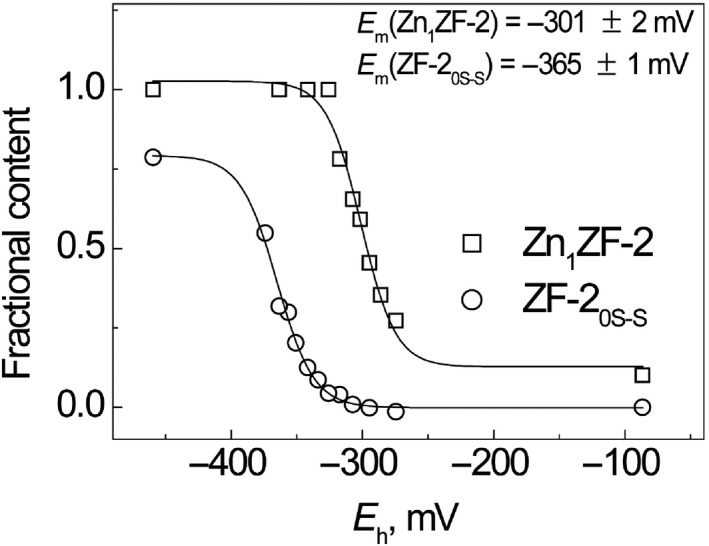
Comparison of redox midpoint potentials of apoZF‐2 and Zn_1_ ZF‐2 in BME/BME_ox_ redox buffers. Fractional content of Zn_1_ ZF‐2 (□) and ZF‐2_0S–S_ (○) at different environmental redox potential values generated by 50 mm BME/BME_ox_. Conditions: 5 μm ZF‐2, 75 mm ammonium formate, pH 7.5, 40 °C. The lines are fitted curves.

Figure 2A shows the determination of the redox potential *E*_m_ of Zn_1_ZF‐1 at pH 7.5 from the dependence of fractional content of Zn_1_ZF‐1 in the mixture of apo‐ZF‐1_1S–S_ and Zn_1_ZF‐1 at equilibrium against *E*_h_ of BME/BME_ox_ redox buffer; its value was −284 ± 1 mV (pH 7.5 and 25 °C) (Fig. 4, squares). The midpoint redox potential of Zn_1_ZF‐2 was also determined from the dependence of fractional content of Zn_1_ZF‐2 in the mixture of the apo‐ZF‐2_2S–S_ and Zn_1_ZF‐2 at equilibrium from the redox potential of BME/BME_ox_ redox buffers (Fig. 2B). The data fitted well to Eqn (4), which demonstrates that oxidation of the disulfide bonds in the peptide molecule occurs cooperatively. The fitting yielded a midpoint redox potential value equal to −301 ± 2 mV (pH 7.5 and 40 °C) (Fig. 5).

## Discussion

The midpoint redox potential values for peptides and small proteins, which contain multiple Cys residues, can be determined from the data of direct measurements of average masses of the mixtures of reduced and oxidized compounds by ESI‐MS 22, 23, for larger proteins indirect methods with chemical modification of SH groups should be used 28. The BME/BME_ox_ redox buffer is the most suitable redox buffer for direct mass measurements, since BME does not decrease the efficiency of the ionization of the peptides in the ESI process 23. The standard redox potential for BME is similar to that of GSH (−253 mV for BME and −262 mV for GSH) 26, 29 and the redox potential range of this buffer can be increased by changing the buffer concentration.

Table 1 summarizes the midpoint redox potential values for the apo and Zn(II)‐bound forms of ZF‐1 and ZF‐2 peptides at pH 7.5. It can be concluded that the Cys_2_His_2_ type of ZF (ZF‐1) has a more positive *E*_m_ value compared with the Cys_4_ type of ZF (ZF‐2), which speaks for their lower tendency to form disulfide in the cellular environment. Disulfides in the Cys_4_ type of ZF (ZF‐2) form cooperatively and require higher reducing capacity for reduction. In order to understand the biological significance of differences of midpoint redox potential values of ZFs they should be compared with the redox potential values in cellular conditions. The redox potential value in the cytosol of eukaryotic cells, determined *in situ*, is approximately −350 mV (pH 7.5) 16, 30, 31, 32, 33. Thus, the apo forms of the Cys_2_His_2_ type of ZFs are predominantly reduced in these conditions, whereas the Cys_4_ type of ZFs is substantially (approx. 70%) oxidized. However, both reduced and oxidized forms of apo‐ZFs are apparently present in cellular conditions.

**Table 1:** Comparison of the measured midpoint redox potentials for apo and Zn‐containing forms of ZF‐1 and ZF‐2 at pH 7.5

	*E*_m_ (mV)	
	ZF‐1	ZF‐2
Apo peptide	−326	−365
Zn_1_ peptide	−284	−301
Δ*E*_m_	42	64

Binding of Zn(II) induced a substantial shift in the redox potential value of both ZF peptides (42 mV for ZF‐1 and 64 mV for ZF‐2 at pH 7.5) indicating that Zn(II) peptides are more resistant to oxidation as compared with apo‐peptides. As the process of the oxidation of the zinc forms involves the release of Zn(II) ions, the protection from oxidation should depend on the affinity of ZF towards Zn(II). The larger shift in the *E*_m_ value in the case of ZF‐2 is in agreement with the observation that Cys_4_ ZFs tend to have higher affinity towards Zn(II) ions than the Cys_2_His_2_ ZFs 34, 35.

*E*_m_ values for Zn(II) forms of both types of ZF peptides are similar indicating that under normal cellular conditions the Zn(II) forms of ZFs are preferably reduced, but even a small shift in the environmental redox potential could result in partial oxidation of the ZF peptides and release of Zn(II) ions, which indicates that ZF proteins might function as redox switches. Such a switching might occur during cellular differentiation and proliferation, which are connected with substantial changes towards more oxidative cellular redox potential values 15. Zn(II) ions protect ZF proteins from oxidation in cellular conditions and support their functioning. Both studied ZF peptides are part of the proteins that are involved in cell division, proliferation and apoptosis and their regulation by environmental redox conditions might play an important role in their functioning.

## Author contributions

PP and JS designed the research. JS and EK performed the experiments. JS, EK, PP and VT analyzed the results; the manuscript was written with contributions from all authors. All authors gave their approval to the final version of the manuscript.
